# Heterometallic Molecular Complexes Act as Messenger Building Units to Encode Desired Metal-Atom Combinations to Multivariate Metal–Organic Frameworks

**DOI:** 10.1021/jacs.2c06142

**Published:** 2022-08-12

**Authors:** Clara López-García, Stefano Canossa, Joke Hadermann, Giulio Gorni, Freddy E. Oropeza, Víctor A. de la Peña O’Shea, Marta Iglesias, M. Angeles Monge, Enrique Gutiérrez-Puebla, Felipe Gándara

**Affiliations:** ‡ Materials Science Institute of Madrid − Spanish National Research Council (ICMM-CSIC), Calle Sor Juana Inés de la Cruz 3, 28049 Madrid, Spain; ∥ EMAT, Department of Physics, University of Antwerp, Groenenborgerlaan 171, 2020 Antwerp, Belgium; § CELLS-ALBA Synchrotron, carrer de la Llum 2-26, 08290, Cerdanyola del Vallès, Barcelona Spain; ⊥ Photoactivated Processes Unit IMDEA Energy Institute, Móstoles Technology Park, Avenida Ramón de la Sagra 3, Móstoles, Madrid 28935, Spain

## Abstract

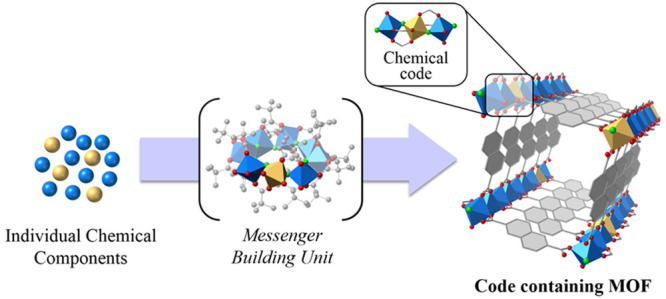

A novel synthetic approach is described for the targeted preparation of multivariate metal–organic frameworks (MTV-MOFs) with specific combinations of metal elements. This methodology is based on the use of molecular complexes that already comprise desired metal-atom combinations, as building units for the MTV-MOF synthesis. These units are transformed into the MOF structural constituents through a ligand/linker exchange process that involves structural modifications while preserving their originally encoded atomic combination. Thus, through the use of heterometallic ring-shaped molecules combining gallium and nickel or cobalt, we have obtained MOFs with identical combinations of the metal elements, now incorporated in the rod-shaped secondary building unit, as confirmed with a combination of X-ray and electron diffraction, electron microscopy, and X-ray absorption spectroscopy techniques.

Creating solids that contain specific chemical information in the form of precise combinations and sequences of various chemical elements remains a major synthetic challenge. Reticular chemistry provides design rules for creating materials such as metal–organic frameworks (MOFs) with desired topological features through the assembly and repetition of secondary building units (SBUs)^1^ and organic linkers. Moreover, multiple chemical constituents can be combined within a MOF ordered backbone.^2−6^ However, exerting control over their disposition is still uncommon,^7−9^ making it difficult to devise materials with specific atomic sequences. This is due to the numerous possibilities for metal distributions in the SBUs of multivariate (MTV) multimetal MOFs,^10−12^ including those constructed from rod-shaped SBUs,^13^ where multiple scenarios for cation distribution are possible.^11^ In addition, the one-pot incorporation of specific metal combinations is not always achievable because their simultaneous introduction in a given SBU might not be compatible with the synthetic procedures for a targeted compound.^14^ Postsynthetic metal-exchange^15^ is an alternative approach for replacing metal cations, but this method is also restricted to certain SBUs and metal elements. During multimetal MTV-MOF reaction formation, the incorporation of multiple metal elements must have compatible incorporation kinetics to avoid the formation of individual, single-metal products or to achieve homogeneous distributions along the crystals. Therefore, alternative synthetic methods to extend the range of metal combinations with precise control are highly significant. Bearing this in mind, we hypothesized that by using molecular entities that already contain specific metal combinations, it might be possible to create multimetal MTV-MOFs with encoded atomic sequences and ratios that otherwise cannot be achieved with traditional methodologies. Hence, we propose a novel synthetic approach based on the use of heterometallic molecular species to produce multimetal MTV-MOFs with desired metal combinations. Molecular precursors have already been successfully used for obtaining single-metal MOFs.^16,17^ Consequently, it is reasonable to think that molecular species that already contain specific combinations of different metal elements might be transformed into MOF SBUs upon reaction with organic linkers, while maintaining the initially selected metal combinations. Noting that these molecular units will carry desired chemical information encoded in the form of a specific metal-atom combination, from the molecular to the reticular state, but without preserving their initial shape and composition, we envisage them as messenger building units (*m*BU) (Figure 1). Our strategy to successfully implement this novel approach is based on the use of molecular compounds able to incorporate multiple metal elements with coordination environments compatible with those expected in the MOF. Thus, they must be formed with anionic ligands, which can be exchanged by the selected MOF linkers.

**Figure 1:**
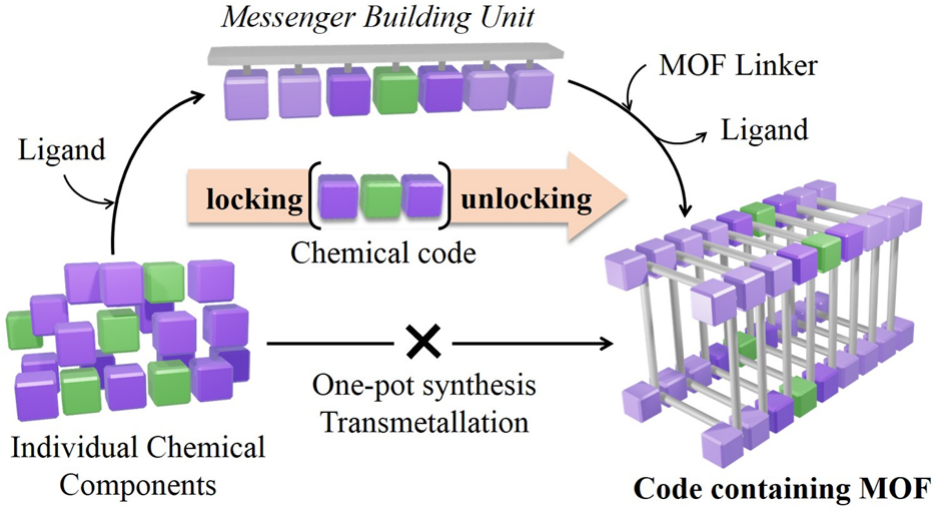
In the *m*Bu approach, different chemical elements are assembled intp a molecular species, locking a specific chemical code, which is preserved through transformation of this unit and MOF formation.

They must also be isolated in solid form to allow their use as reactants while at the same time be labile to allow their transformation through a ligand/linker exchange process during the MOF formation. With these considerations, we have focused on the well-known family of heterometallic ring-shaped clusters, which can be prepared with different combinations of tri- and divalent metal elements.^18^ In particular, we have synthesized the gallium–nickel heterometallic ring with formula [Ga_7_NiF_8_(PivO)_16_], Ga7Ni-*m*BU from now on, [PivOH = pivalic acid, (CH_3_)_3_CCO_2_H]. By following reported procedures,^19^ we have isolated this compound to be used for screening of MOF synthetic conditions. Single-crystal X-ray diffraction experiments (SCXRD) confirmed the formation of the expected ring-shaped molecule.^19^ The metal atoms are in an octahedral environment, coordinated to pivalate and fluorine anions, and with the presence of dipropylammonium cations inside the ring, for charge balance (Figure 2a). Since gallium and nickel atoms cannot be distinguished with SCXRD, the presence of nickel is confirmed with energy dispersive X-ray spectroscopy (EDS) measurements carried out with different crystals, whose results supported the 7:1 ratio expected for the insertion of one nickel atom per molecular unit (Figure S1).

**Figure 2:**
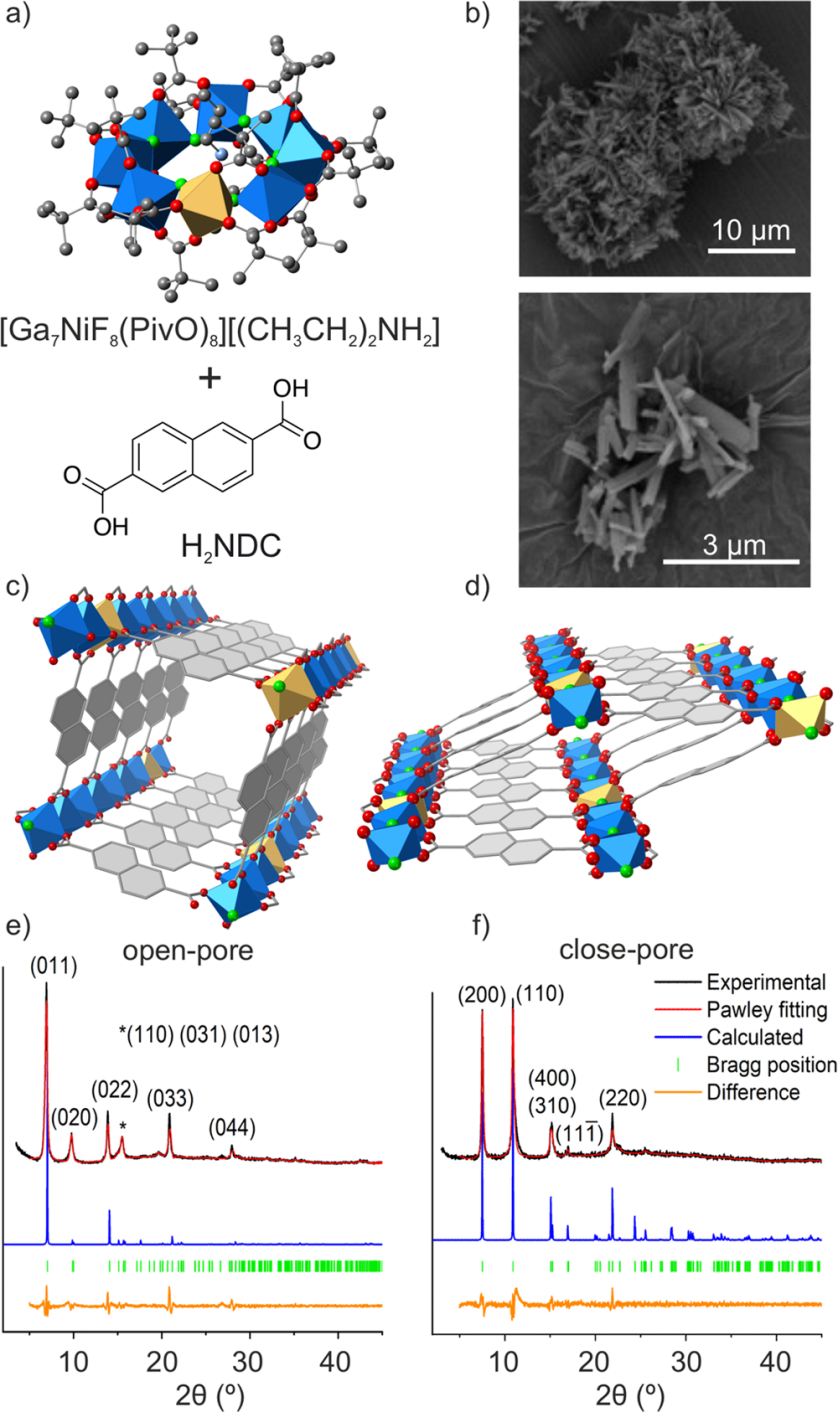
(a) Representation of the selected heterometallic molecular complex to be used as *m*BU. (b) SEM images of Ga7Ni-MIL-69 crystals. (c, d) Ga7Ni-MIL-69 open- and close-pore forms, respectively, and (e, f) comparison of the experimental PXRD patterns with the ones calculated for the open- and close-pore structures. Blue: gallium, gold: nickel, carbon: gray, oxygen: red, fluor: green.

Following, MOF synthesis experiments were carried out by heating a DMF solution containing the selected Ga7Ni-*m*BU and the organic linker 2,6-napthalenedicarboxylic acid (H_2_NDC) at 180 °C for 72 h (Supporting Information S1.3). A microcrystalline powder was obtained. The NMR spectrum of an acid digested sample confirmed the incorporation of NDC linkers and almost complete disappearance of pivalate anions, demonstrating the successful exchange process (Figure S3). SEM images show a homogeneous particle morphology, which consists of needle-like crystals (Figure 2b). All attempts to grow crystals sufficiently large to complete SCXRD experiments were unsuccessful. Nonetheless, powder X-ray diffraction (PXRD) experiments demonstrated the crystallinity of the product. Moreover, we found that upon drying of the sample, it undergoes a structural transformation, with marked differences in the position of the diffraction peaks. This change is reversible, and the original pattern can be recovered after immersion of the sample in fresh DEF (Figure S4), strongly indicating framework flexibility as responsible for the changes in the pattern. Following, electron diffraction experiments were conducted to obtain additional structural information. Despite the presence of prominent diffuse intensities due to lattice strain and disorder, indexing of 3D electron diffraction data indicates an orthorhombic cell, with tentative lattice parameters *a* = 5.2 Å, *b* = 6.4 Å, *c* = 19.1 Å (S2.9). These values are consistent with a closed-pore conformation of MIL-69 (PARPII CSD code).^20^ This structure has been previously reported with the combination of gallium and NDC, and is also known to have a flexible framework with open and close pore forms. Two crystalline models were built up based on MIL-69 open and close forms (S2.2), and their PXRD patterns were compared to the experimental ones, showing an excellent agreement (Figure 2c–f). From this structural analysis, we can confirm that a MOF isoreticular to MIL-69 has been successfully obtained with the use of the Ga7Ni-*m*BU, herein denoted Ga7Ni-MIL-69. The structure of MIL-69 consists of rod-shaped SBUs, formed by octahedral trivalent atoms sharing vertices, which in the present case should be occupied by fluorine atoms already present in the Ga7Ni-*m*Bu. To ensure that nickel is incorporated in the MOF, EDS analyses were carried out on the samples. The results confirmed that indeed the newly formed MOF is made up of both metal elements, and furthermore the output ratio is in agreement with the initial 7:1 of the molecular complex. CHN and TXRF elemental analysis are consistent with the proposed formula [Ga_7_NiF_8_(NDC)_8_]·3DMF·5H_2_O (S1.3). Since the ^1^H NMR spectrum of digested Ga7Ni-MIL-69 did not show a significant presence of dipropylammonium to balance the charge produced by the presence of divalent nickel, this can be compensated for by protonation of one carboxylic acid or by replacement of a fluorine atom by a water ligand per each eight metal atoms. To further confirm that the inorganic SBUs comprise both elements, and exclude the possibility that nickel atoms are in the form of adsorbed or deposited chemical species, an X-ray spectroscopy study was completed. X-ray photoelectron spectroscopy clearly shows the presence of Ni in Ga7Ni-MIL-69 (Figure S5). The Ni 2 p region has a peak at 857.6 eV, which is significantly higher than the peak position commonly found in Ni oxide compounds (854–856 eV)^21^ and close to the reported peak position for NiF_2_, 858.12 eV.^22^ This observation suggests the presence of a direct Ni–F bond in the structure of the MOF, which is further clarified below. Thus, an X-ray absorption spectroscopy study was also completed at the Ni and Ga K edges to comprehensively characterize their chemical local environments (S2.4).

Figure 3 shows the XANES spectra for the MOF sample and reference materials. The position of the Ni K edge corresponding to Ga7Ni-MIL-69 (8346 eV) indicates the presence of octahedral Ni^2+^ species. Similarly, the Ga K-edge XANES spectrum is also consistent with the presence of only one type of coordination for Ga^3+^. Moreover, the edge position of the MOF is ∼2 eV higher than *β-*Ga_2_O_3_, which is correlated with the presence in the coordination sphere of both fluorine and oxygen, as opposed to just oxygen, consistent with the transformation of the *m*BU. Through the comparison of the Ga and Ni K edge spectra shifted by the corresponding edge values (E0) (Figure S6), very similar XANES features, including a white line and a broad resonance at 60 eV above the edge, are clearly visible, suggesting a similar local structure for Ga^3+^ and Ni^2+^. In addition, by comparing the EXAFS spectra, Figure 4, similar k^2^χ(k) signals are observed for Ni and Ga in the sample with different oscillation frequencies due to different bond distances. The Fourier transform confirms the similarity of the local structure around Ga^3+^ and Ni^2+^, with the main contribution being an intense M–O first shell (M = Ga, Ni). A second intense shell, associated with M–M distance, is observed for NiO and β-Ga_2_O_3_ but not for the MOF, ruling out the presence of metal oxide species.

**Figure 3:**
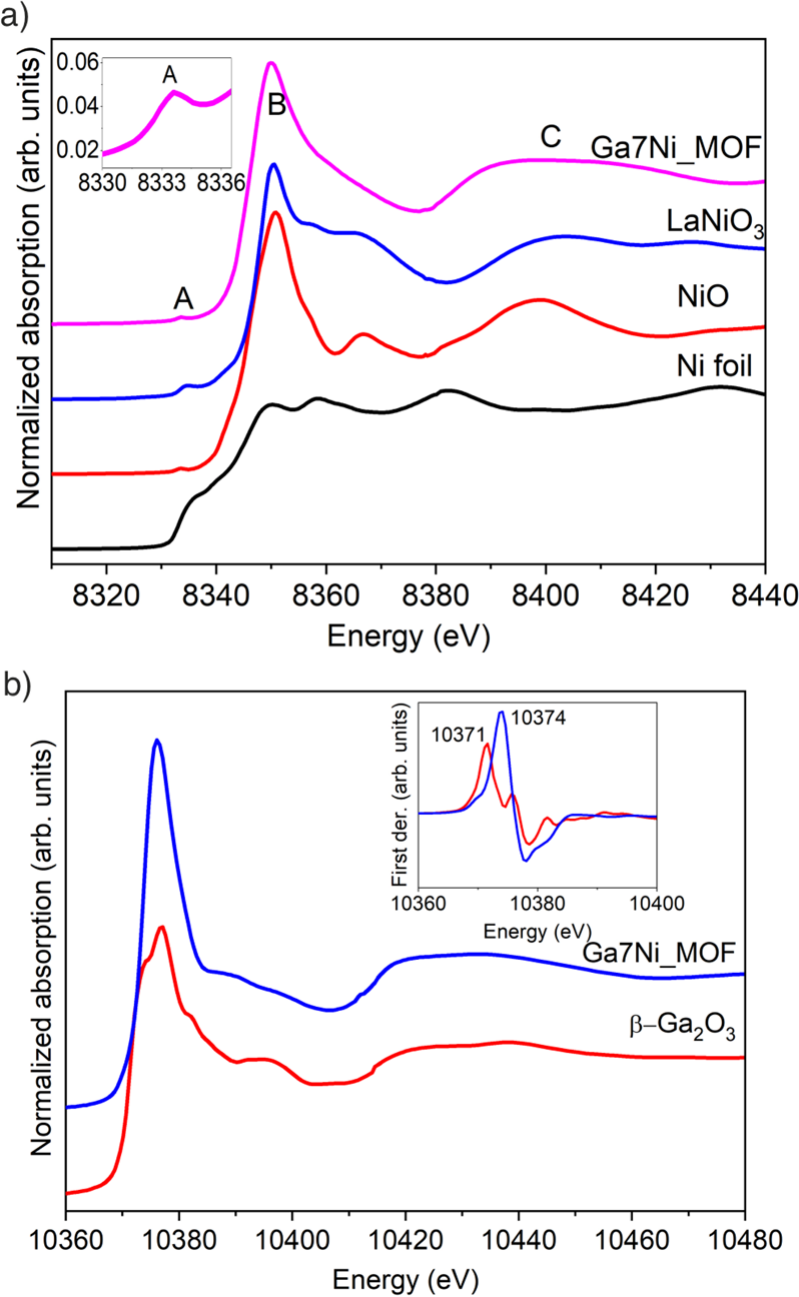
(a) Ni K edge XANES spectra of MIL-69(Ga,Ni) with reference compounds. The inset shows detail of the 1*s*-3*d* prepeak transition, (b) Ga K edge XANES spectra of Ga7Ni-MIL-69 and β-Ga_2_O_3_. The inset shows the first derivative spectra.

**Figure 4:**
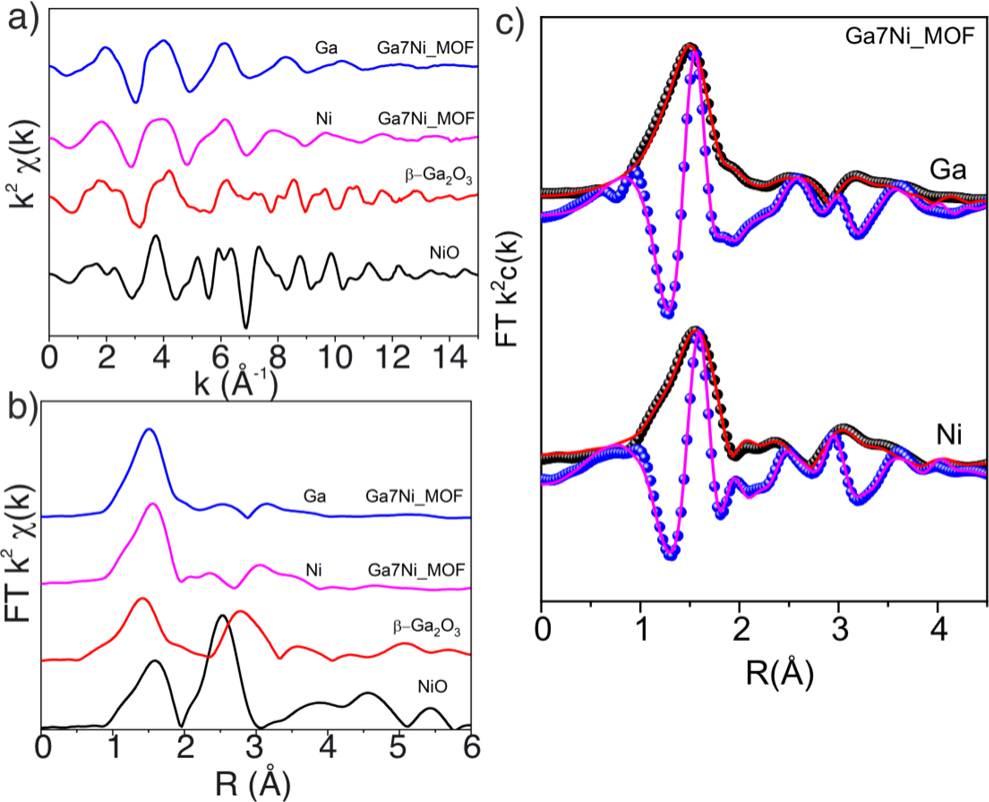
(a) Ni and Ga K edge k^2^χ(k) signals, (b) Fourier transform (FT) magnitude of k^2^χ(k) signals of Ga7Ni-MIL-69 and references, (c) magnitude (black spheres) and real part (blue spheres) of FT k^2^ χ(k) with the corresponding fit (solid line).

Fitting of EXAFS data was performed in the *R*-range of 1–4 Å (in the scale without phase correction) to have a structural model for both ions. We employed the crystal structure corresponding to the above-described MIL-69 framework, now with the presence of both nickel and gallium atoms at the SBUs. Only the most intense and single scattering paths were considered to fit the data. Figure 4b shows the fit of magnitude and real part FT k^2^χ(k) for both Ni and Ga. Good agreement is obtained between the data and fit using the same model for the local structure around both ions, indicating that Ni^2+^ and Ga^3+^ occupy the same sites. In particular, a Ga–Ga or Ni–Ga scattering path was necessary to fit the shell around 3 Å (Table S2, Figure S7). While we note that there are several ways in which the Ga7Ni *m*BU can be transformed in the MOF SBU depending on how it is cleaved, from the XAS data we can confirm that nickel sites remain isolated between gallium atoms in the MOF, thus preserving the chosen chemical code. Moreover, this would also be preserved even if two rod SBUs are not identical to each other, like in other reported multimetal MTV-MOFs.^11^ Encouraged by the successful use of a *m*BU for programming a new chemical combination in a MOF structure, we aimed at demonstrating that other atomic codes can be introduced with this strategy. Thus, we synthesized the equivalent Ga7Co-*m*BU based on the analogous molecular compound [Ga_7_CoF_8_(pivalate)_16_]. The obtained crystalline product, Ga7Co-MIL-69, is isostructural to Ga7Ni-MIL-69, as demonstrated by the coincidental PXRD pattern (Figure S8), and incorporation of cobalt and gallium at expected ratios was also proven by EDS (Figure S2). Control synthesis experiments were carried out by using mixtures of the corresponding gallium and nickel or cobalt salts as a metal precursor, in combination with H_2_NDC. However, in all cases, only gallium MIL-69 was obtained, without the presence of any of the divalent metal elements, suggesting that the joint incorporation of the two cations might not be feasible following a one-pot approach. Preliminary studies with the use of other ditopic linkers, such as 1,4-benzenedicarboxylic acid, indicates that our method can be used to obtain other isoreticular MOFs with the same programmed metal combinations. Thus, an analogous Ga7Ni-MIL-53^23^ material was obtained, displaying the same 7:1 metal ratio (S3).

In view of these results, we believe that the use of *m*BUs for the synthesis of multimetal MTV-MOFs will open up new opportunities to accomplish the incorporation of desired atomic combinations previously inaccessible with traditional procedures. We anticipate that this will be useful for achieving high precision in the incorporation of isolated metal sites in porous solids or to create highly controllable environments with modulable electronic or magnetic states, which is potentially relevant to fields such as heterogeneous catalysis or quantum computing.

## Supporting Information Available

Experimental details, including synthesis procedures, PXRD patterns, EDS spectra, ^1^H NMR, XPS, and XAS analysis, thermograms, sorption isotherms, and electron diffraction analysis (PDF)
