# Supplementary material for: Heterometallic Molecular Complexes Act as Messenger Building Units to Encode Desired Metal-Atom Combinations to Multivariate Metal–Organic Frameworks
Source: J Am Chem Soc. 2022 Aug 12;144(36):16262–6. doi: 10.1021/jacs.2c06142 (PMC9479064; doi:10.1021/jacs.2c06142)
Supplement: Supplementary file 1 — ja2c06142_si_001.pdf [file ja2c06142_si_001.pdf]

Supplementary Information

Heterometallic molecular complexes act as messenger building units to encode desired metal-atom combinations to multivariate metal-organic frameworks.

Clara López-García,[‡] Stefano Canossa,[⁑†] Joke Hadermann,[⁑] Giulio Gorni,[§] Freddy E. Oropeza,[ø] Víctor A. de la Peña O'Shea,[ø] Marta Iglesias,[‡] M. Angeles Monge,[‡] Enrique Gutiérrez-Puebla,[‡] Felipe Gándara[‡]

‡ Materials Science Institute of Madrid – Spanish National Research Council (ICMM-CSIC). Calle Sor Juana Inés de la Cruz 3, 28049, Madrid, Spain

⁑ EMAT, Department of Physics, University of Antwerp, Groenenborgerlaan 171, 2020 Antwerp, Belgium

§CELLS-ALBA Synchrotron, carrer de la Llum 2-26, 08290, Cerdanyola del Vallès, Barcelona, Spain

ø Photoactivated Processes Unit IMDEA Energy Institute, Móstoles Technology Park, Avenida Ramón de la Sagra 3, Móstoles, Madrid, 28935, Spain.

SUPPLEMENTARY INFORMATION

S1. STARTING MATERIALS AND SYNTHESIS 1

S1.1. Materials and reagents 1

S1.2. Synthesis of Heterometallic Rings (HMR) Ga7M (M= Ni, Co). 1

S1.3. Synthesis of Ga7M-MIL-69 (M= Ni, Co). 4

S2. CHEMICAL AND STRUCTURAL Ga7M-MIL-69 (M= Ni, Co) CHARACTERIZATION 5

S2.1. ^{1}H-NMR 5

S2.2. Crystal structure models 6

S2.3. X-Ray Photoelectron Spectroscopy (XPS) 8

S2.4. X-Ray Absorption Spectroscopy (XAS) 9

S2.5. Ga7M-MIL-69 (M= Ni, Co) powder X-ray diffraction comparative 10

S2.6. Thermal-Stability Analysis 11

S2.7. Scanning Electron Microscopy (SEM) 11

S2.8. Textural Characterization 13

S2.9. Electron diffraction analysis 14

S3. SYNTHESIS AND CHEMICAL Ga7Ni-MIL-53 CHARACTERIZATION 20

S3.1. Materials and reagents 20

S3.2. Synthesis of Ga7Ni-MIL-53. 20

S3.3. PXRD and SEM-EDS characterization of Ga7Ni-MIL-53. 20

S4. REFERENCES 23

S1. STARTING MATERIALS AND SYNTHESIS

S1.1. Materials and reagents

$GaF_3 \cdot 3H_2O$ (99,5% purity) and di-n-propylamine (99% purity) was purchased from abcr GmbH. $NiCO_3 \cdot 2Ni(OH)_2 \cdot 4H_2O$ (min. 44% Ni) was purchased from Carlo Erba. $CoCl_2 \cdot 6H_2O$ (99,0%) was purchased from Merck. Pivalic acid (PivOH) (>99% purity) was purchased from TCI. 2,6-naphtalenedicarboxylic acid (2,6-NDC) (99% purity) was purchased from Sigma-Aldrich. N,N-Dimethylformamide (DMF) (99.9% purity) and acetonitrile (MeCN) (HPLC grade) were purchased from Labkem. Toluene (>99,7% purity) was purchased from J. T. Baker.

S1.2. Synthesis of Heterometallic Rings (HMR) Ga7M (M= Ni, Co).

HMR-Ga7M synthesis was based on the original previously reported procedure[1]. To prepare the HMR-Ga7Ni molecular complex, a mixture of gallium fluoride trihydrate $GaF_3 \cdot 3H_2O$ (2,0160 g, 11.097 moles), nickel(II) carbonate basic hydrate, $NiCO_3 \cdot 2Ni(OH)_2 \cdot 4H_2O$ (0.3300 g, 0.877 mmol), pivalic acid (20.0000 g, 195.829 mmol) and di-n-propylamine (1.0004 g, 9.8829 mmol) were heated and stirred at 170 ºC under a slow N_2 flow for 18 hours. The dark greenish mixture is cooled to 80 ºC and MeCN (50 mL) is added, after which the mixture is vigorously stirred for 15 minutes. The resulting light green mixture was cooled to room temperature and the microcrystalline product was separated from the waters by centrifugation (10 min, 5200 rpm). Once separated, the product was washed with acetonitrile (3 x 20 mL) and acetone (3 x 20 mL), after which it was left to air dry at room temperature. The obtained solid was recrystallized in pentane to give light green crystals. Yield: 0.7640 g, 67%.

The procedure to prepare the analogous HMR-Ga7Co molecular complex, is similar to the HMR-Ga7Ni, adding $CoCl_2 \cdot 6H_2O$ (0.6340 g, 2.638 mmol), instead of the nickel salt. The post-synthetic procedure to purify the solid is similar to the previous one described below, obtaining in this case pink crystals. Yield: 0.4417 g, 21%.

Table S1. Crystalographic parameters comparative between the already reported **HMR-Ga7M** (M= Ni, Co)[1], and the analogues synthesized for this work.

Crystalographic parameters	Reported HMR Ga7Ni	HMR Ga7Ni	Reported HMR Ga7Co	HMR Ga7Co
Crystal system	Monoclinic	Monoclinic	Triclinic	Monoclinic
Space group	$P2_1/c$	$P2_1/c$	$P\bar{1}$	$C2/c$
a (Å)	25,041(2)	25,2979(11)	16,4095(12)	26,94(2)
b (Å)	16,621(3)	16,9278(8)	16,4309(12)	20,807(16)
c (Å)	31,514(4)	31,6290(13)	26,131(2)	26,51(2)
Volume (Å^3)	12821(3)	13306,5(10)	6401,38(90)	13870(19)
α (^{0})	90	90	73,942(5)	90
β (^{0})	102,172(9)	100,761(2)	73,998(5)	111,06(3)
γ (^{0})	90	90	75,933(1)	90
Goodness-of-fit on F^2	0,955	1,065	0,720	1,042
R_1 [all data]	0,0942	0,1440	0,2973	0,1239
wR_2 [all data]	0,1491	0,3074	0,2193	0,2836

a)

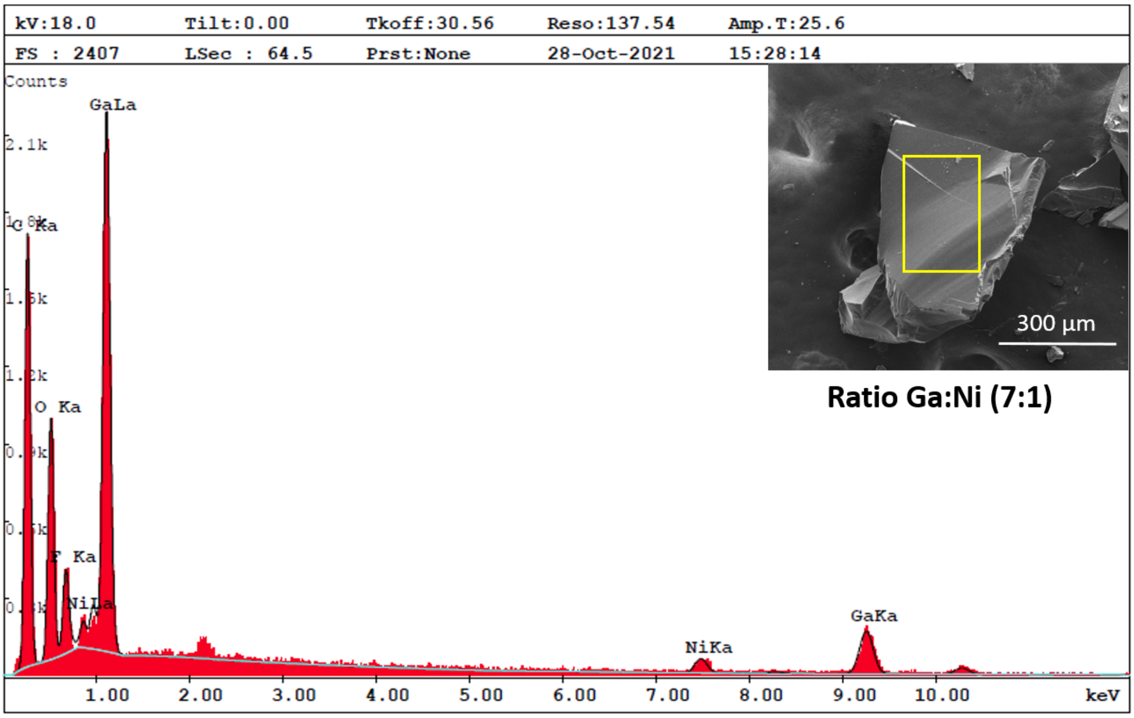

b)

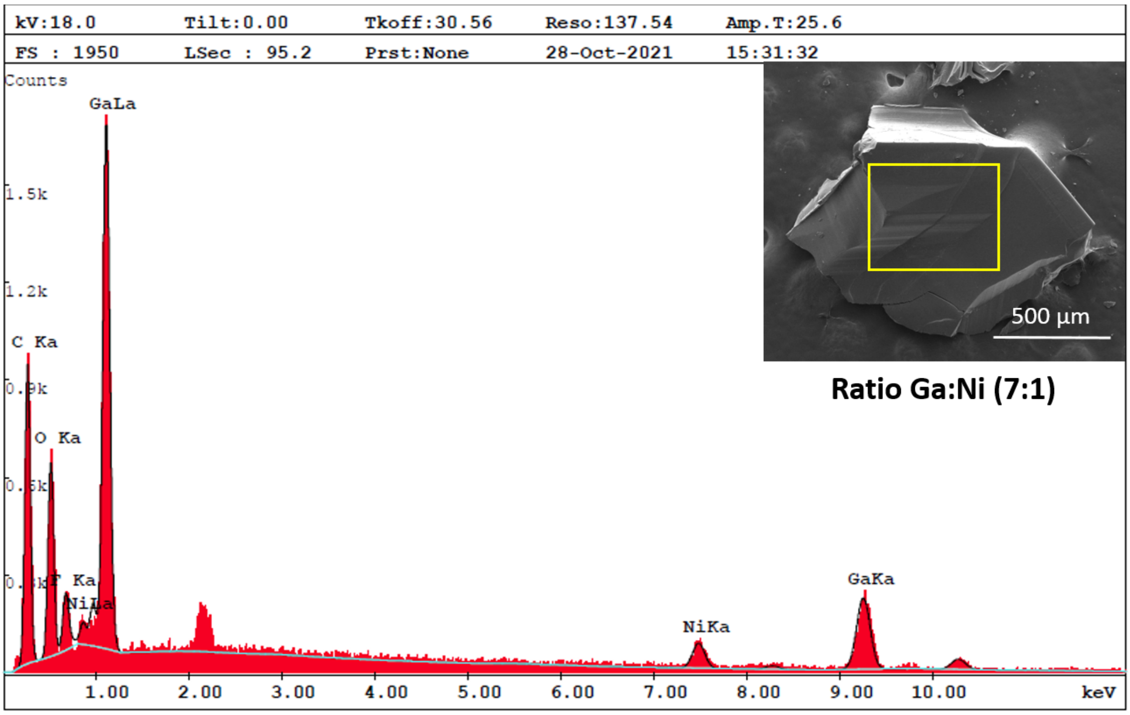

c)

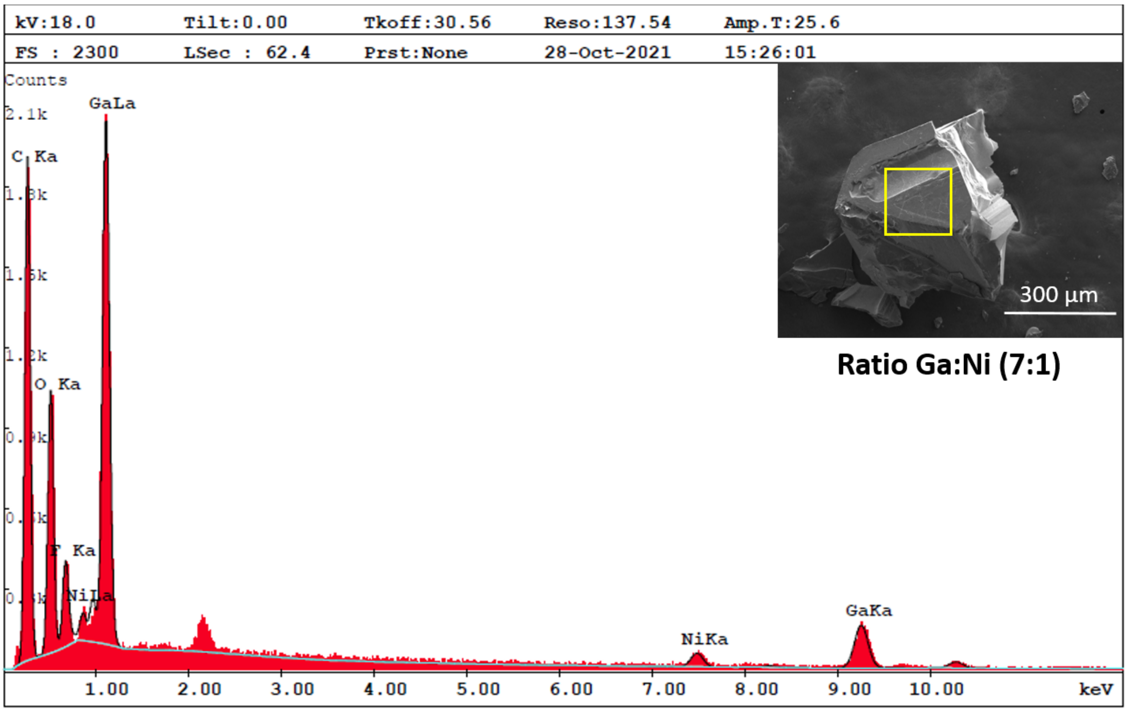

Figure S1. a-c) EDX analysis and SEM images for different HMR Ga7Ni crystals.

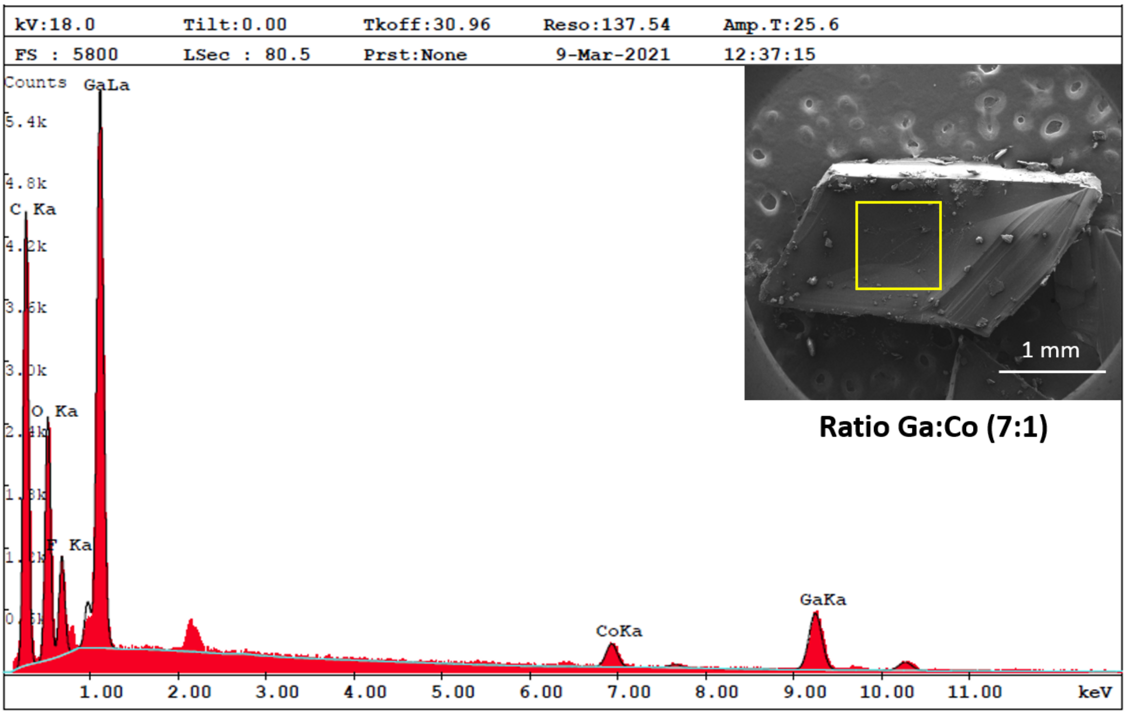

Figure S2. EDX analysis and SEM image for HMR Ga7Co.

S1.3. Synthesis of Ga7M-MIL-69 (M= Ni, Co).

For the Ga7M-MIL-69 synthesis, firstly 5 mL of toluene were added to HMR-Ga7M (0.0500 g, 0.021 mmol), previously placed into a 50 mL teflon solvothermal reactor, and stirred for 5 minutes. Meanwhile, 10 mL of DMF were added to 2,6-H_2NDC (0.0723 g, 0.331 mmol), sonicated for 4 minutes and stirred 1 more minute before the solution were added to the HMR-Ga7M mixture. It was placed in the stove and kept at 180 ºC for 72 hours.

The obtained beige microcrystalline product is insoluble in water and common organic solvents, and was separated by centrifugation (10 min, 5200 rpm) and washed with DMF (3 x 5 mL), after which the final product was stored under clean DMF at room temperature. Yield: 30.9 mg, 54%.

CHN and TXRF elemental analysis are consistent with the proposed formula $[Ga_7NiF_8(NDC)_8]\cdot 3DMF\cdot 5H_2O$: %weight, experimental: C: 46.23, N: 1.42, H: 3.10, Ga: 17.03, Ni: 2.67, calculated: C: 46.05, N: 1.53, H: 2.94, Ga: 17.82, Ni: 2.14). We note that the presence of divalent nickel atoms in the structure

demands presence of an additional positively charge species. This is provided by a dipropylammonium cation in the molecular complex. However, the ^{1}H NMR spectrum of digested Ga7Ni-MIL-69 did not evidence significant presence of this cation to compensate the charge. While the presence of ammonium species formed by solvent decomposition during MOF synthesis cannot be completely ruled out, the charge could also be compensated by protonation of one carboxylic acid, or by replacement of a fluorine atom by a water ligand per each eight metal atoms.

S2. CHEMICAL AND STRUCTURAL Ga7M-MIL-69 (M= Ni, Co) CHARACTERIZATION

S2.1. ^{1}H-NMR

Proton nuclear magnetic resonance (^{1}H-NMR) spectra were recorded using a Bruker AVANCE II spectrometer, with a 500 MHz frequency, for liquid samples.

To prepare the ^{1}H-NMR sample, 10.0 mg of MIL-69(Ga,Ni), were dissolved in a mixture of 0.5 mL of DMSO-d_6 and 10 μL of DCl (35% wt. in D_2O).

In the resulting NMR spectrum (**Figure S3**), the observed signals correspond to the 2,6-NDC aromatic protons, and the DMF aldehyde and aliphatic protons.

Based on this results, the presence of pivalate groups and di-n-propylamine could be discarded, suggesting the complete substitution of the pivalates groups with the 2,6-NDC ligand.

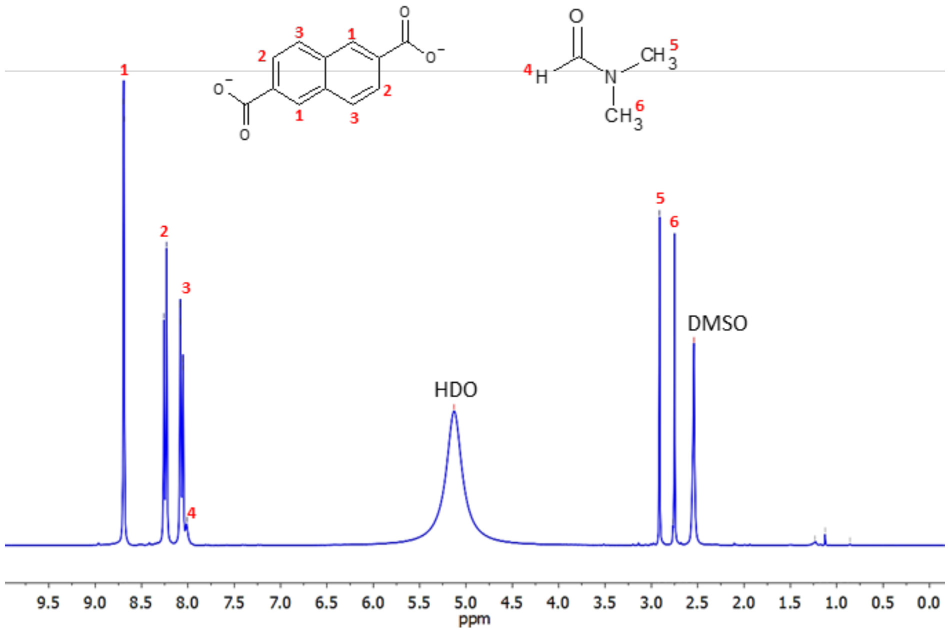

Figure S3. ^{1}H-NMR of MIL-69(Ga,Ni) in DMSO and DCl (35% wt. in D_2O).

S2.2. Crystal structure models

Two crystalline models were built up based on MIL-69 open and close forms, and their PXRD patterns compared to the experimental ones. The open pore form corresponds to a monoclinic cell with *I2/a* space group, and lattice parameters a = 6.245 Å, b = 17.910 Å, c = 17.797 Å, β = 97.05º. Conversely, the close pore form crystallizes in the *C2/c* space group with cell parameters a = 23.505 Å, b = 8.654 Å, c = 6.809 Å, β = 90.74º, which are consistent with the lattice determined through electron diffraction, after a transformation from orthorhombic to monoclinic system. The variation in the symmetry can be attributed to the different conditions for data acquisition between electron diffraction and PXRD, in particular the high-vacuum conditions employed for the earlier. The atomic fractional coordinates for the optimized structures are the following:

Open pore form

Atom	x	y	z
O1	-0.59388	-0.06754	0.42981
C2	-0.78449	-0.08937	0.40322
C3	-0.80563	-0.14565	0.34212
C4	-0.99934	-0.15223	0.29326

C5	-0.86804	-0.30695	0.16794
O6	-0.54245	-0.43521	0.0729
H7	-0.47321	-0.18775	0.3708
H8	-0.36512	-0.384	0.20042
C9	0.34761	0.74704	0.77452
C10	-0.01911	0.70478	0.73485
H11	-0.16884	0.70791	0.69716
Ga1	-0.5	0	0.5
F1	-0.75	0.03024	0.5

Close pore form

Atom	x	y	z
O1	-0.06612	0.11399	-0.09848
O2	-0.05238	0.14459	-0.42758
C3	-0.14954	0.19632	-0.3246
C4	-0.16939	0.23203	-0.52062
C5	-0.24749	0.29239	-0.76381
C6	-0.0854	0.15168	-0.27658
C7	-0.22885	0.25637	-0.57177
C8	-0.19394	0.18405	-0.18629
H9	-0.13778	0.2426	-0.64583
H10	-0.21539	0.30392	-0.88738
H11	-0.18392	0.15435	-0.02768
F1	0	-0.07147	-0.25
Ga1	0	0	0

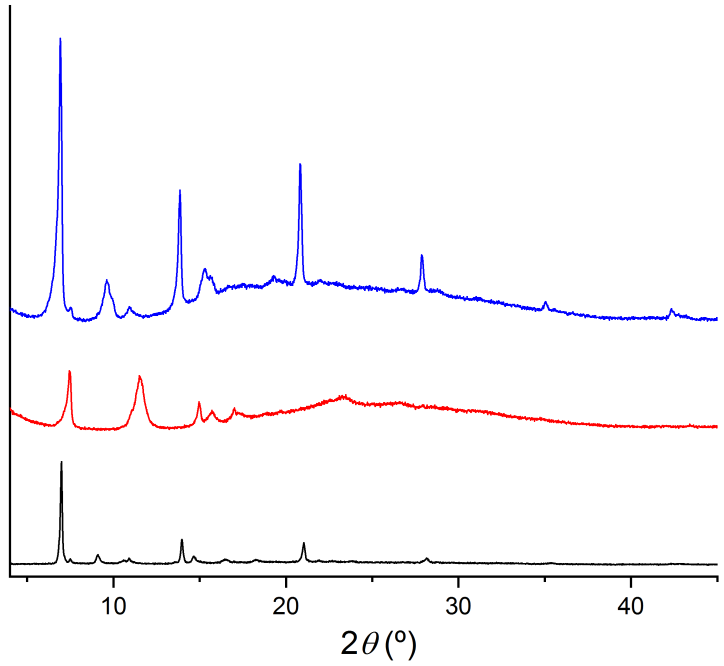

Figure S4. Comparison of the experimental PXRD patterns from the Ga7Ni-MIL-69 as synthesized (black), after activated under vacuum at 100 °C, 24 h (red) and the activated solid after being submerged in DEF for 4 days (blue). Sample after solvent exchange shows minor presence of close-pore form, probably due to partial sample drying during pattern acquisition.

S2.3. X-Ray Photoelectron Spectroscopy (XPS)

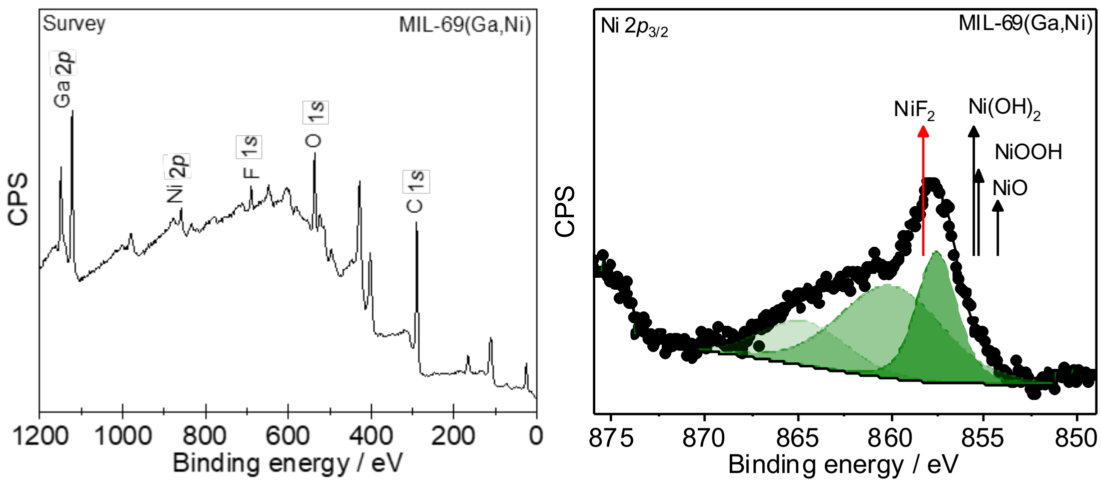

Figure S5. Survey XPS of Ga7Ni-MIL-69 and high resolution scan in the Ni 2p region. Reported peak positions for different Ni oxides and NiF_2 are indicated in the plot.[2]

S2.4. X-Ray Absorption Spectroscopy (XAS)

Ga and Ni K-edge X-ray absorption spectra were measured at the CLÆSS beamline of the ALBA synchrotron using a Si(311) monochromator. Higher harmonics rejection was achieved by a collimating and focusing mirror tuned at specific angles and with proper coatings. The GaNi samples were prepared as 5 mm pellets and the XAS spectra collected in transmission mode at room temperature under vacuum conditions and with an incident energy resolution below 0.3 eV. The beam size was 100 x 300 μm and the incident and transmitted intensities were measured with two ionization chambers. Ni foil, NiO, $LaNiO_3$ and β-Ga_2O_3 were also measured and used as references for energy calibration and data analysis. Data analysis was carried out by ATHENA and ARTEMIS software of the DEMETER package.[3] XANES simulation was performed with the FDMNES software.[4]

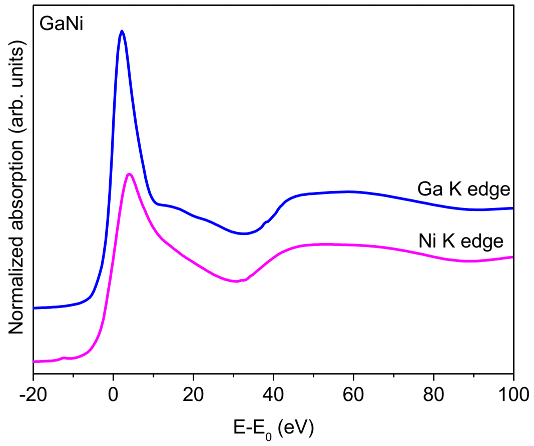

Figure S6. Ni and Ga K edge XANES spectra of GaNi MOFs. The spectra were shifted by E_0 value of 8346 and 10374 eV, respectively.

Table S2. Result of EXAFS fit. N is the coordination number, R the bond distance and σ^2 the Debye-Waller parameters.* indicates the coordination number fixed for the fit according to the CIF file of the Ga MOF.

Absorber	Atom	N	R (Å)	σ^2(10^{-3} Å^{-2})	R-factor
Ni	O	6.3±0.6	2.03±0.01	6±1	0.006
	C	4*	2.95±0.03	10±4	
	Ga	2*	3.37±0.02	7±2	
	O	8*	4.38±0.05	7±5	
Ga	O	6.4 ±0.5	1.95±0.01	8±1	0.008
	C	4*	3.02±0.04	15±7	
	Ga	2*	3.40±0.02	10±2	
	O	8*	4.38±0.04	13±5	

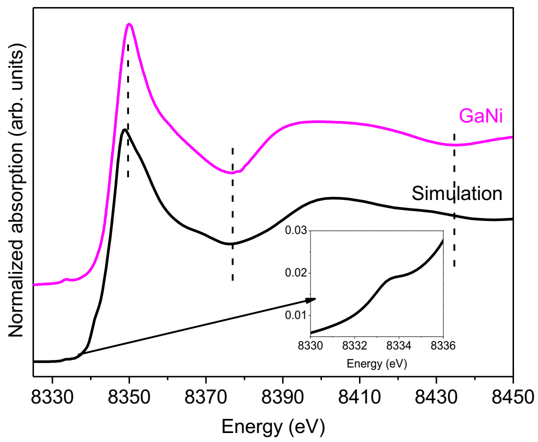

Figure S7. Comparison of measured and simulated Ni K edge spectra. The vertical dashed lines are a guide for the eye. The inset shows a detail of the pre-edge transition at 8333 eV reproduced in the simulation.

S2.5. Ga7M-MIL-69 (M= Ni, Co) powder X-ray diffraction comparative

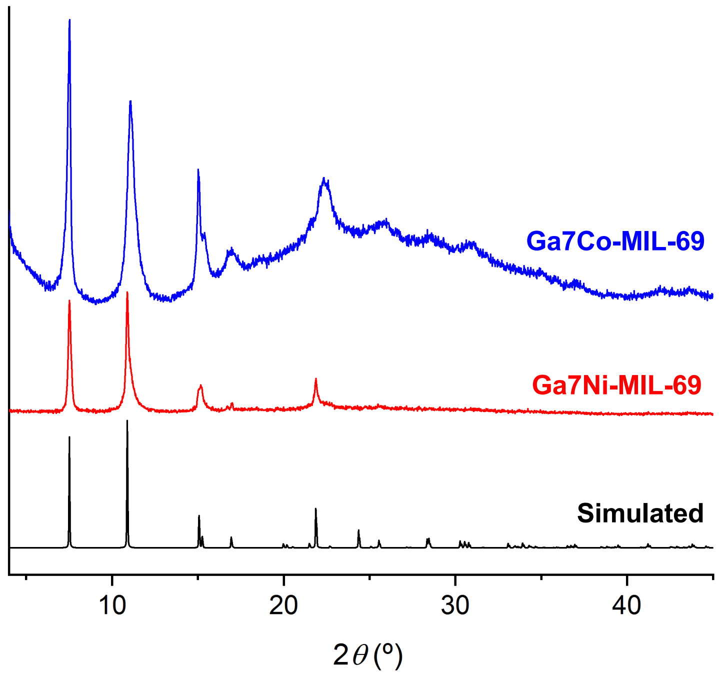

Figure S8. Comparison of the experimental PXRD patterns with the calculated from the open model. Blue, red and black difractograms represent Ga7Co-MIL-69, Ga7Ni-MIL-69 and the open model, respectively.

S2.6. Thermal-Stability Analysis

Thermogravimetric experiments for MIL-69(Ga,Ni) were performed in a STA ATD/DSC/TG Q600 from TA Instruments under an air steam with a ramp of 10 ºC/min between 25 and 800 ºC (**Figure S9**).

The experiment shows two main weight losses. The first one of 8.589%, at 296 ºC, which corresponds to the evaporation of DMF molecules occluded in the framework pores, and a second one of 72.16%, at 548 ºC, due to the degradation of the material, caused by the linker decomposition.

At this temperature the organic ligands are lost by the decarbonilation of SBU, resulting on a nickel gallium oxide ($NiGa_2O_4$) residue.

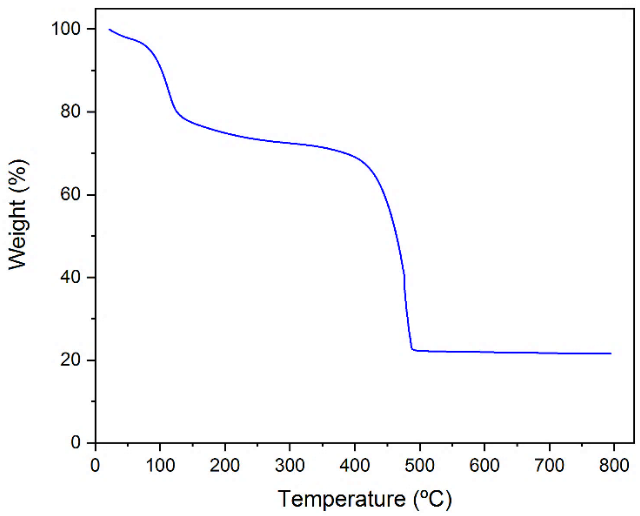

Figure S9. Thermogravimetrical analysis of MIL-69(Ga,Ni) under an air stream.

S2.7. Scanning Electron Microscopy (SEM)

The SEM images were taken with a Philips XL30 S-FEG microscope. The samples were placed on a carbon double sided tape, attached to an aluminum sample holder, and later metallized with a layer of gold (13 nm thick).

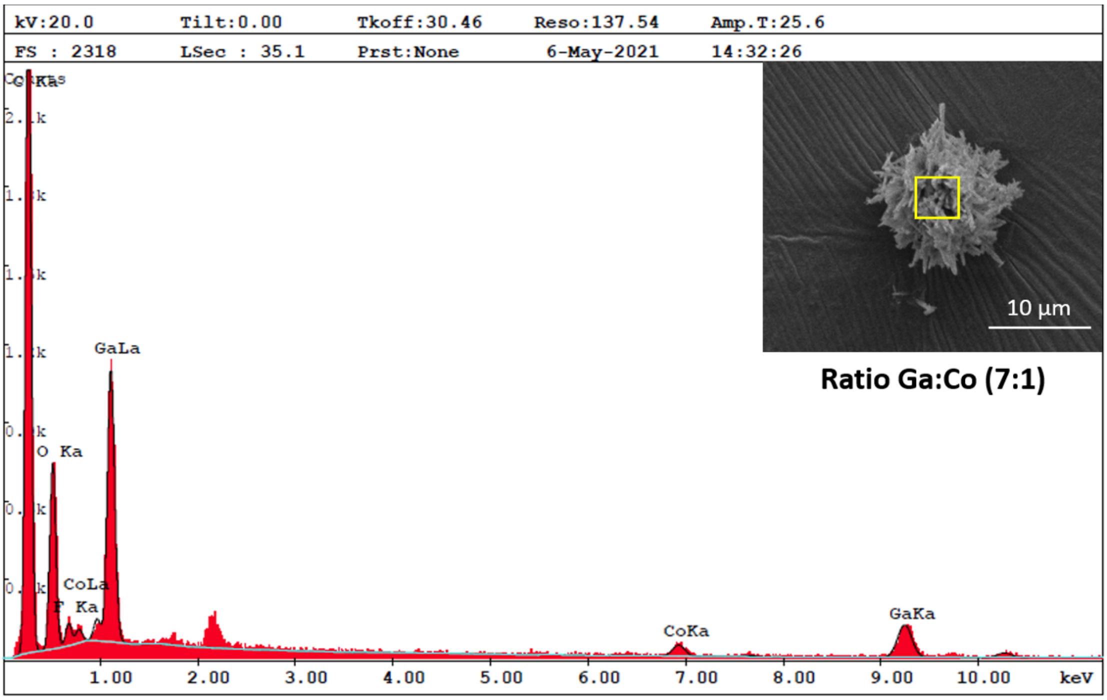

Figure S10. EDX analysis and SEM image for Ga7Co-MIL-69.

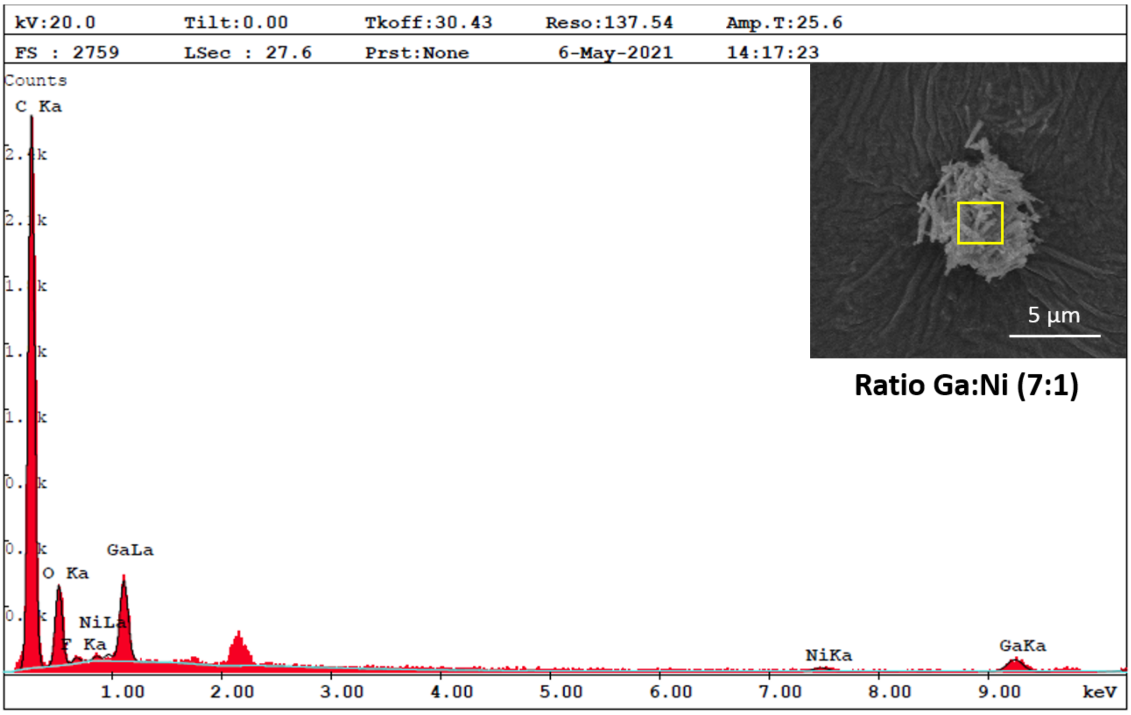

Figure S11. EDX analysis and SEM image for Ga7Ni-MIL-69.

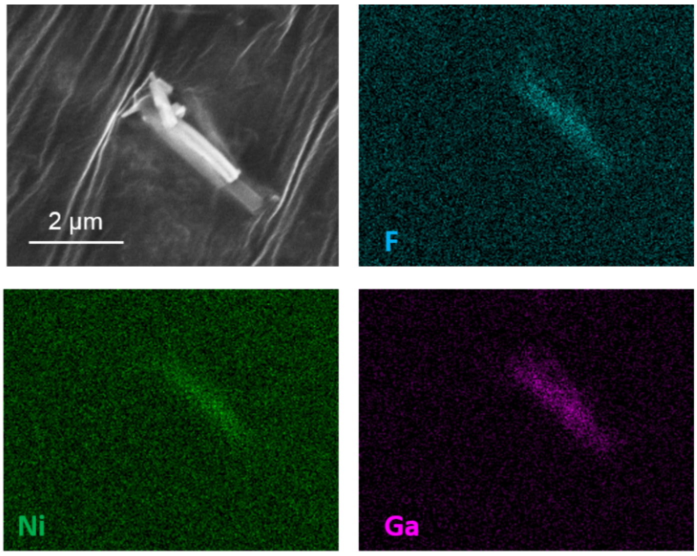

Figure S12. EDS mapping corresponding to an isolated particle of Ga7Ni-MIL-69, showing the presence of Ga, F, and Ni in the crystals. The data was collected for activated samples.

S2.8. Textural Characterization

Activated samples were used for collecting CO_2 and N_2 sorption isotherms (**Figure S13 and Figure S14**) at 195 and 77 K, respectively, using a Quantachrome Autosorb-1 instrument and a Micromeritics ASAP2020. Prior to measurement, the sample was degassed overnight at 120 ºC under vacuum.

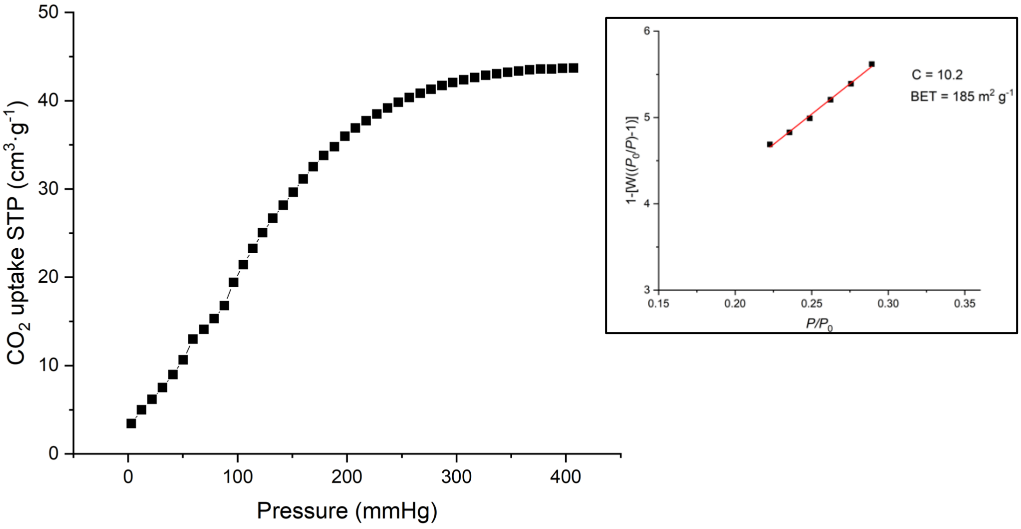

Figure S13. CO_2 adsorption isotherm of Ga7-Ni-MIL-69 collected at 195 K. The calculated pore volume is 0.08 cm^3 g^{-1}

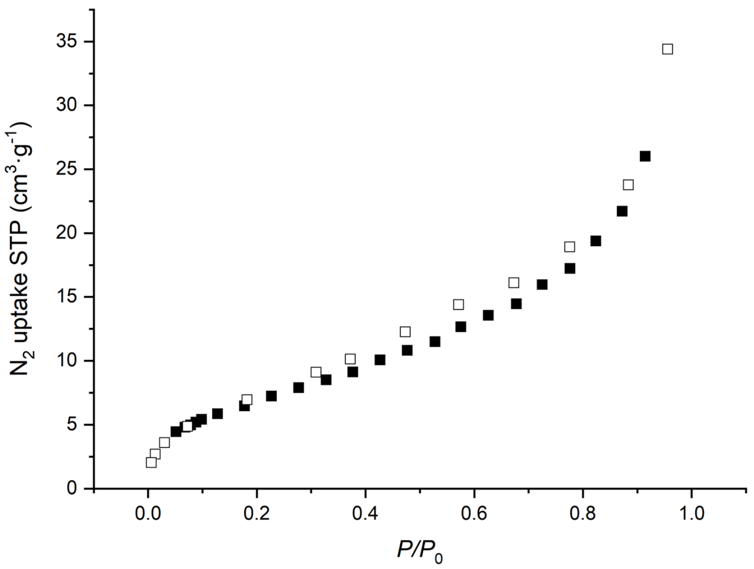

Figure S14. N_2 adsorption-desorption isotherm Ga7-Ni-MIL-69 collected at 77K. Filled and blank squares represent adsorption and desorption points, respectively.

S2.9. Electron diffraction analysis

3D electron diffraction data were collected on a Titan 'cubed' transmission electron microscope operated at 300 kV and at room temperature. Dry polycrystalline powder was gently crushed between two glass slides and transferred on a continuous-carbon copper grid by placing it in contact with the crushed powder. The grid was loaded onto a Fischione 2020 tomography holder and inserted into the TEM without further treatment.

Electron beam defining settings included C2 lens excitation corresponding to spot size nr. 9 and a C2 aperture of 20µm. Data collections were conducted by using a custom-made script allowing for a step-wise tilt and acquisition of diffraction patterns by a Gatan Ultra Scan 1000 CCD camera.

The camera length that was used has been calibrated based on a gold polycrystalline sample, and its Debye rings were used to obtain the size of the detector's pixels in reciprocal space.

Three crystals in total were analyzed with the following measurement parameters:
Crystal 1: tilt step 0.25°, total range 140°, frame time 2 seconds.
Crystal 2: oscillation step 0.50°, total range 140°, frame time 5 seconds.

Crystal 3: oscillation step 0.25°, total range 150°, frame time 3 seconds.

The resulting three datasets were processed by the software pets2 version 2.1.20211012.1037 (Palatinus, L. PETS-program for analysis electron diffraction data. in *PETS-program for analysis electron diffraction data, Institute of Physics of the AS CR: Prague, Czechia, 2011*.) and CrysAlisPro v41.93a (Rigaku Oxford Diffraction, (2017), CrysAlisPro Software System, Oxford, UK.) to confirm the indexing results by using different programs. Complications due to crystal twinning and poor data quality led us to determine unit cell parameters based on the selection and refinement of the primitive triclinic unit cell fitting all reflections, and subsequent check for alternative settings (centering) during the unit cell determination process in pets2. The indexing procedure (from pets2) resulted in the following sets of unit cell metrics:

Crystal 1

Unconstrained parameters (Å, °):

a, b, c, α, β, γ: 5.2 6.4 19.0 90.3 91.0 90.3

Cell volume: 634.7 $Å^3$

Constrained parameters (Å, °):

a, b, c, α, β, γ: 5.3 6.4 19.1 90 90 90

Cell volume: 639.7 $Å^3$

Crystal 2

Unconstrained parameters (Å, °):

a, b, c, α, β, γ: 5.4 6.6 19.0 89.7 89.7 89.6

Cell volume: 676.4 $Å^3$

Constrained parameters (Å, °):

a, b, c, α, β, γ: 5.4 6.7 19.0 90 90 90

Cell volume: 677.3 $Å^3$

Crystal 3

Unconstrained parameters (Å, °):

a, b, c, α, β, γ: 5.3 6.3 19.1 90.6 91.2 90.5

Cell volume: 640.2 $Å^3$

Constrained parameters (Å, °):

a, b, c, α, β, γ: 5.3 6.3 19.1 90 90 90

Cell volume: 643.6 $Å^3$

Below, reciprocal space reconstructions produced by pets2 highlight broad scattering features due to lattice strain and defects, and presence of crystal twinning. The combinations of these effects hampered a quantitative use of the data for structure solution and refinement.

In the following reconstructions, the intensity scale has been adjusted to the range 0-100 counts. For the reconstruction process we used a pixel size of 0.002 $Å^{-1}$ and a slab thickness of 0.014 $Å^{-1}$, and we did not adopt symmetry averaging.

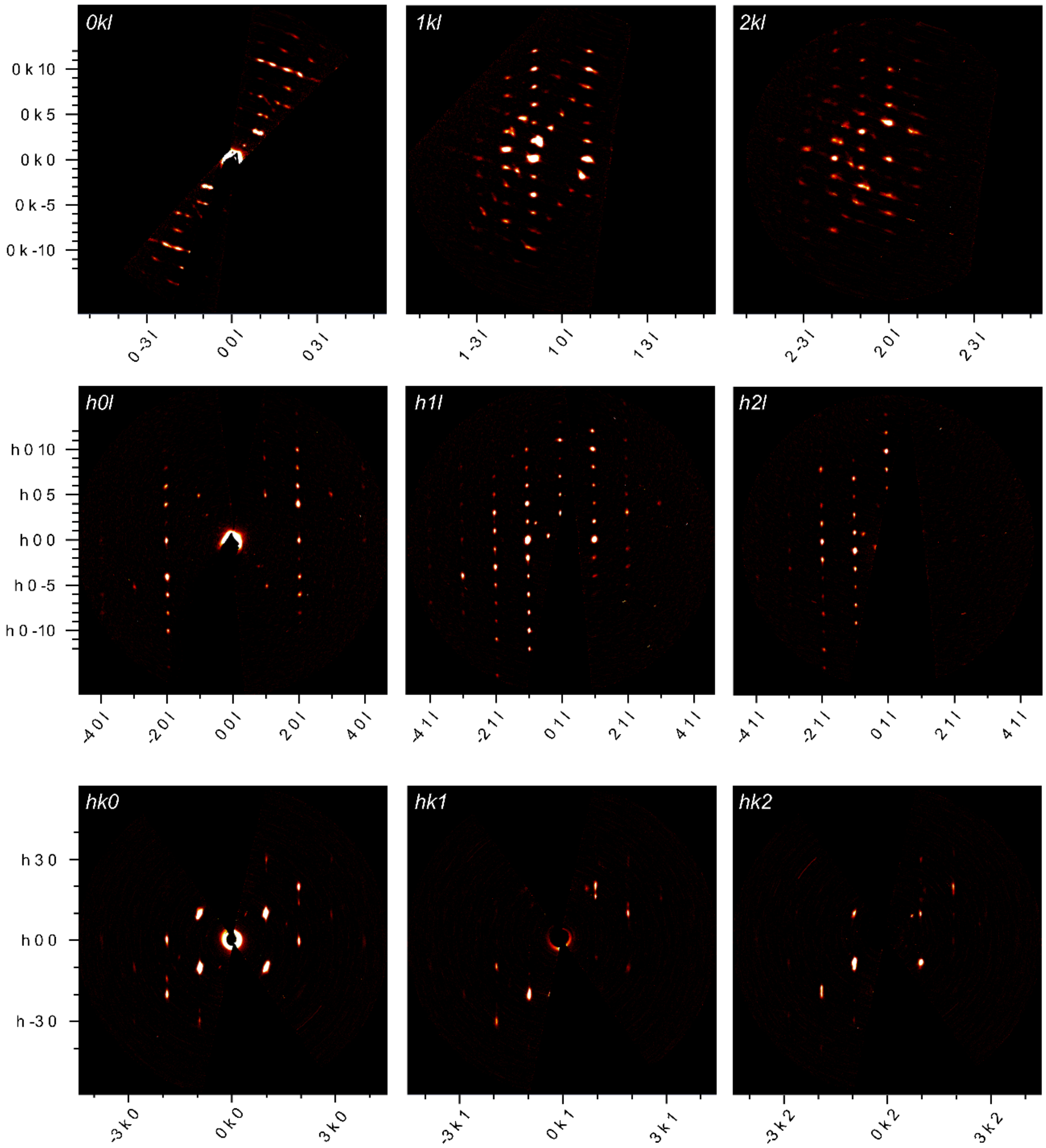

Figure S15. Main reciprocal space slabs for crystal 1.

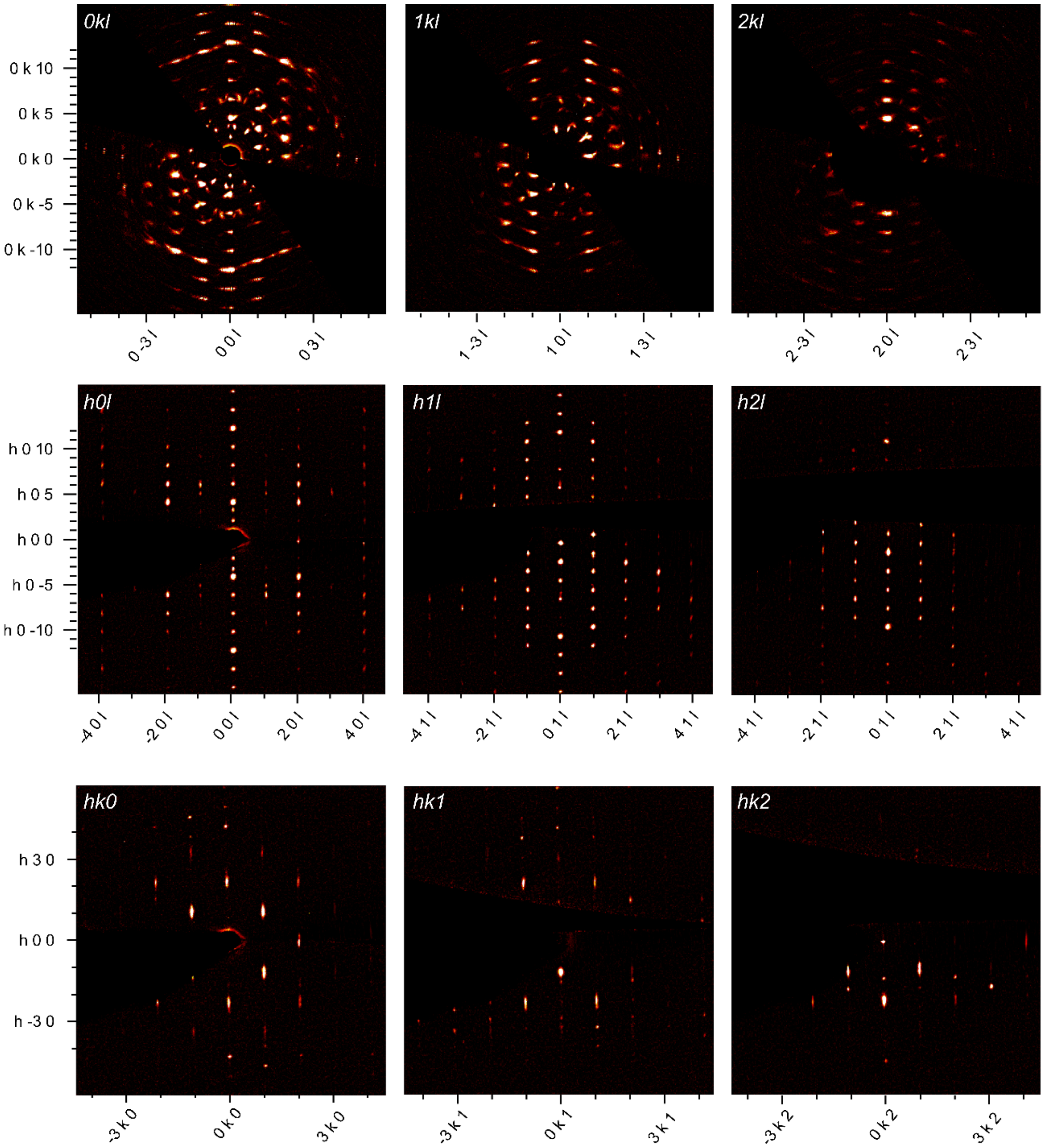

Figure S16. Main reciprocal space slabs for crystal 2.

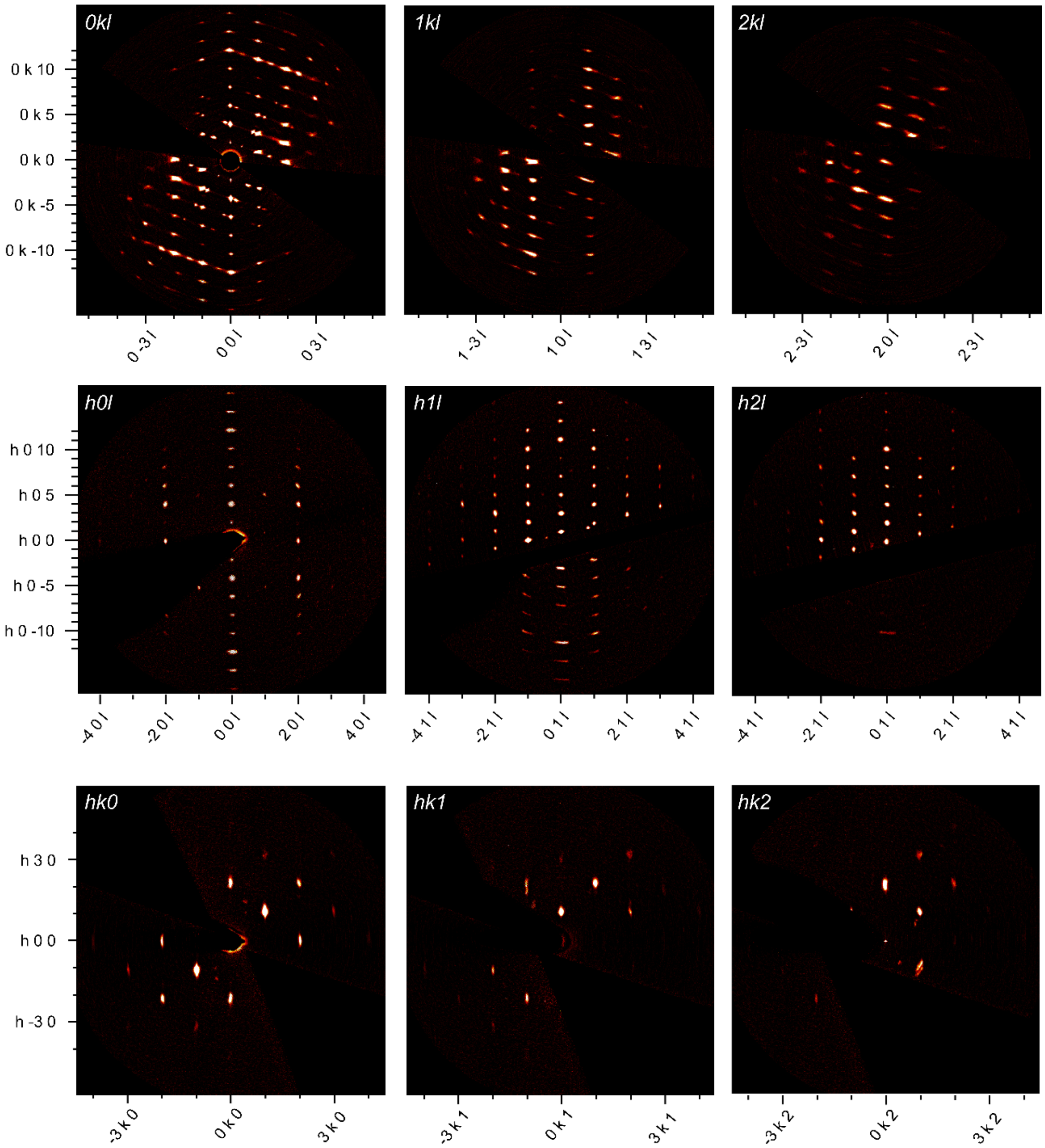

Figure S17. Main reciprocal space slabs for crystal 3.

S3. SYNTHESIS AND CHEMICAL GA7NI-MIL-53 CHARACTERIZATION

S3.1. Materials and reagents

Benzene-1,4-dicarboxylic acid (H_2bdc) (98% purity) was purchased from Alfa Aesar. N,N-Dimethylacetamide (DMA) (99% purity) was purchased from abcr, and ethanol absolute was purchased from Scharlau.

S3.2. Synthesis of Ga7Ni-MIL-53.

For the Ga7Ni-bdc synthesis, HMR-Ga7M (0.0500 g, 0.021 mmol) and H_2bdc (0.0246 g, 0.145 mmol) were placed into a 50 mL teflon solvothermal reactor, and 4 mL of DMA were added to the mixture. It was placed in the stove and kept at 170 ºC for 89 hours.

The obtained beige microcrystalline product was separated by centrifugation (10 min, 5200 rpm) and washed with distilled water and ethanol (3 x 4 mL), after which the final product was stored at room temperature.

S3.3. PXRD and SEM-EDS characterization of Ga7Ni-MIL-53.

A crystal model was built based on the reported MIL-53 with CSD code QOVWOO01[5]. Lattice parameters were adjusted based on the experimental PXRD data, resulting in a $P2_1/c$ monoclinic unit cell with a = 19.480 Å, b = 17.716 Å, c = 6.671 Å and β =103.757°. The structure was geometrically optimized with energy minimization procedures with the use of the Forcite module of Materials Studio. The simulated and experimental PXRD patterns are shown in figure S17.

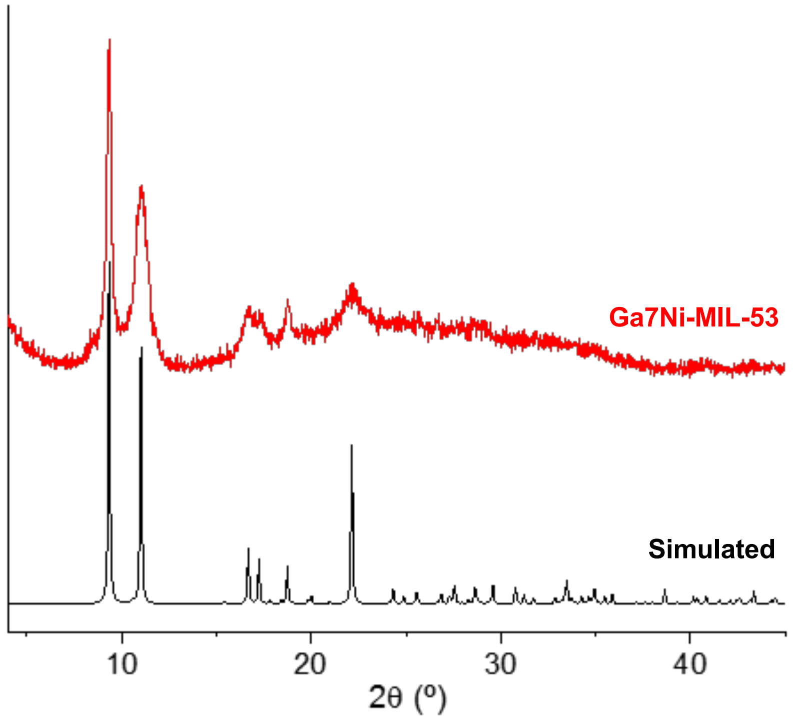

Figure S18. Comparison of the experimental PXRD pattern (red) with the diffractogram for the calculated Ga7Ni-MIL-53 model (black).

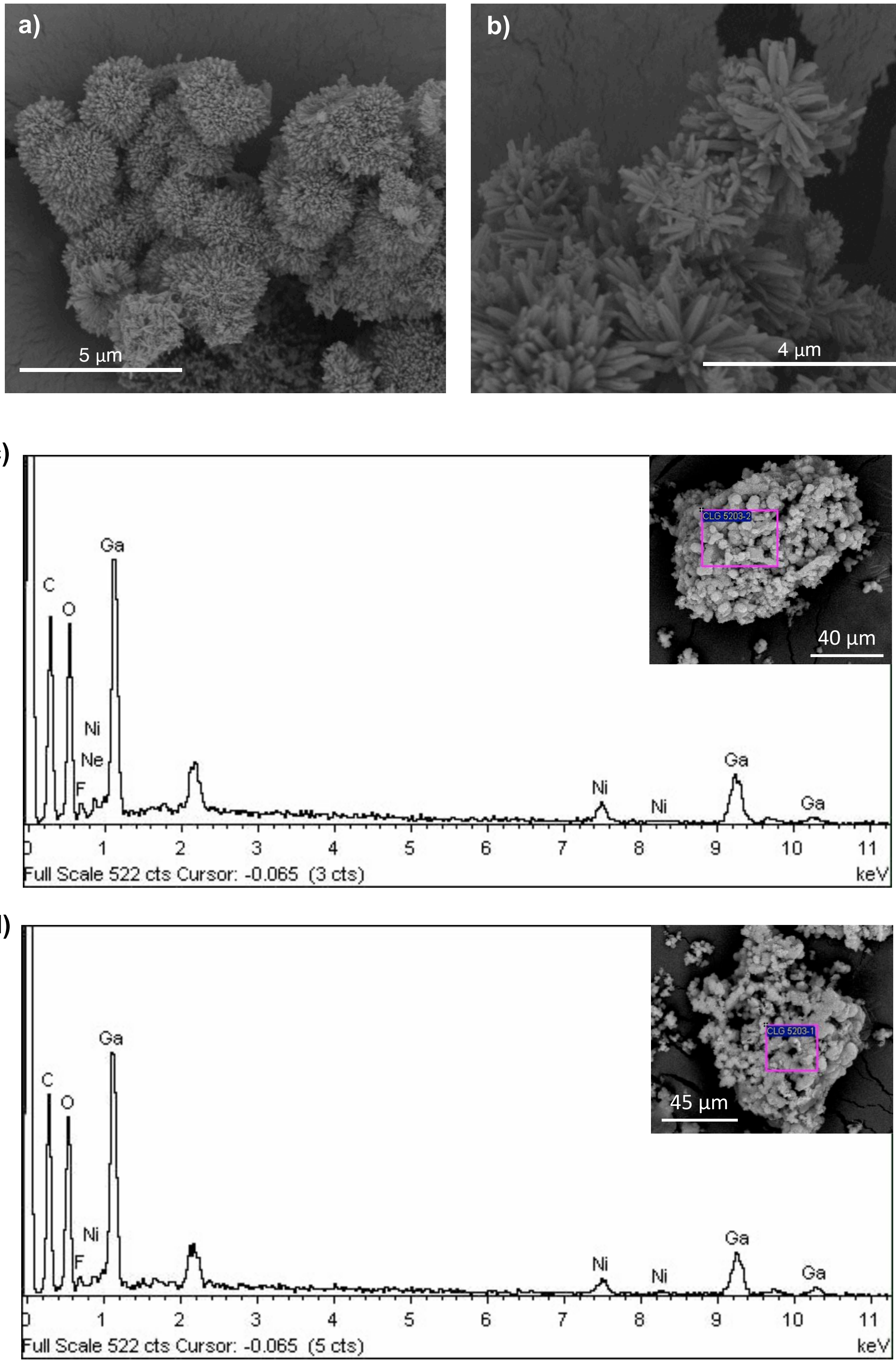

Figure S19. SEM images (a,b) and EDS analysis (c,d) for Ga7Ni-MIL-53.

S4. REFERENCES

(1) Sañudo, E. C.; Muryn, C. A.; Helliwell, M. A.; Timco, G. A.; Wernsdorfer, W.; Winpenny, R. E. P. Al, Ga and in Heterometallic Wheels and Their by-Products. *Chem. Commun.* **2007**, No. 8, 801–803. https://doi.org/10.1039/b613877b.

(2) Biesinger, M. C.; Lau, L. W. M.; Gerson, A. R.; Smart, R. S. C. The Role of the Auger Parameter in XPS Studies of Nickel Metal, Halides and Oxides. *Phys. Chem. Chem. Phys.* **2012**, *14* (7), 2434–2442. https://doi.org/10.1039/c2cp22419d.

(3) Ravel, B.; Newville, M. ATHENA, ARTEMIS, HEPHAESTUS: Data Analysis for X-Ray Absorption Spectroscopy Using IFEFFIT. *J. Synchrotron Radiat.* **2005**, *12* (4), 537–541. https://doi.org/10.1107/S0909049505012719.

(4) Bunău, O.; Joly, Y. Self-Consistent Aspects of x-Ray Absorption Calculations. *J. Phys. Condens. Matter* **2009**, *21* (34). https://doi.org/10.1088/0953-8984/21/34/345501.
